# Divergent Trends in Hospitalizations and Mortality From Diabetes and Hypertension: A Population‐Based Time‐Series Study With Implications for Public Health Nursing in Southern Brazil

**DOI:** 10.1111/phn.70168

**Published:** 2026-08-03

**Authors:** Gabriel Pavinati, Marcos Vinícius Lopes, Aliny de Lima Santos, Mariana Aleixo Terci, Pedro Augusto Bossonario, Edileuza de Fátima Rosina Nardi, Lucas Vinícius de Lima

**Affiliations:** ^1^ Department of Nursing State University of Maringá Maringá Paraná Brazil; ^2^ Department of Medicine State University of Maringá Maringá Paraná Brazil; ^3^ Department of Planning Municipal Health Department Maringá Paraná Brazil

**Keywords:** diabetes mellitus, hospitalization trends, hypertension, mortality trends, public health nursing, time‐series

## Abstract

**Objective:**

To analyze temporal trends in hospitalization and mortality rates due to diabetes mellitus and hypertension in the state of Paraná, Brazil.

**Design:**

Population‐based ecological time‐series study.

**Sample:**

Residents of the state of Paraná, Southern Brazil, from 2014 to 2024.

**Measurements:**

Annual hospitalization and mortality rates per 100,000 inhabitants due to diabetes mellitus and hypertension were calculated using national health information systems and stratified by sex and age group.

**Analytic Strategy:**

Joinpoint regression models were applied to estimate annual and average annual percent changes and identify temporal inflection points.

**Results:**

Hospitalization rates declined for both conditions across most population groups, particularly among older adults. In contrast, mortality rates increased over much of the study period, especially among younger and middle‐aged adults, with stabilization only in recent years. Divergent trends between hospitalizations and mortality were consistent across sex and age strata.

**Conclusions:**

Declining hospitalization rates do not necessarily indicate improved population health outcomes. The coexistence of reduced hospitalizations and increased mortality highlights gaps in early diagnosis, continuity of care, and longitudinal management of chronic conditions. These findings underscore the need for public health nursing strategies that integrate surveillance data, strengthen primary care follow‐up, and address upstream determinants of chronic disease outcomes.

**Abbreviations**
AAPC: Average annual percent change
APC: Annual percent change
CI: Confidence interval
DM: Diabetes mellitus
ICD‐10: International Classification of Diseases, 10th Revision
RECORD: Reporting of Studies Conducted using Observational Routinely‐Collected Data.
SIH: Hospital Information System of the Unified Health System (*Sistema de Informações Hospitalares do* *Sistema Único de Saúde*)
SIM: Mortality Information System (*Sistema de Informações sobre Mortalidade*)
SUS: Unified Health System (*Sistema Único de Saúde*)

## 1 Introduction

Noncommunicable chronic diseases (NCDs), particularly diabetes mellitus (DM) and hypertension, are among the leading causes of morbidity and mortality worldwide and in Brazil (Yezli et al. 2021; Hoq et al. 2023; Leitão et al., 2024). Characterized by a prolonged clinical course and the need for continuous care, these conditions are considered ambulatory care–sensitive conditions, as appropriate management at the primary care level can prevent clinical deterioration and related complications (Neves et al. 2021).

Within the Brazilian Unified Health System (*Sistema Único de Saúde*, SUS), core attributes of primary health care—first‐contact access, longitudinality, care coordination, and comprehensiveness—are essential for addressing DM and hypertension (Macedo et al. 2025). These attributes support risk identification, ongoing clinical monitoring, coordination across levels of care, and the implementation of preventive actions, thereby contributing to the reduction of avoidable hospitalizations and deaths (Macedo et al. 2025).

Despite advances in prevention, screening, and follow‐up of NCDs, substantial challenges remain in the diagnosis and management of DM and hypertension (Neves et al. 2021). In Brazil, for example, underdiagnosis of DM is estimated at approximately 42.5%, exceeding 70% in the Northern region, which hampers timely care within health services (Muzzy et al. 2021). Delayed diagnosis and inadequate management contribute to increased hospitalizations and preventable mortality (Neves et al. 2021; Ferreira et al. 2021).

According to the 2019 National Health Survey, the self‐reported prevalence of DM and hypertension among Brazilian adults was 9.2% (Muzzy et al. 2021) and 23.9% (Julião et al. 2021), respectively. In 2023, data from the Surveillance of Risk and Protective Factors for Chronic Diseases by Telephone Survey indicated even higher prevalence estimates in Curitiba, the capital of Paraná state, with 9.8% for DM and 25.4% for hypertension (Brasil 2025a).

Regarding mortality, national epidemiological reports showed a mortality rate of 26.8 deaths per 100,000 inhabitants due to DM in Brazil in 2021, whereas Paraná presented higher rates than the national average (Brasila 2022). For hypertension, mortality has increased steadily nationwide over the last decade. Beyond mortality, DM and hypertension are associated with reduced quality of life, increased risk of cardiovascular and metabolic complications, and substantial health system costs (Neves et al. 2021; Ferreira et al. 2021; Brasil 2025a, 2025b; Borges et al. 2023).

Given the high burden of these conditions, their impact on population health, and recent national efforts to strengthen primary health care, it is essential to understand how hospitalization and mortality patterns have evolved over time. Therefore, this study aimed to analyze temporal trends in hospitalization and mortality rates due to DM and hypertension in the state of Paraná, Brazil, from 2014 to 2024.

## 2 Materials and Methods

### 2.1 Research Design

This ecological time‐series study was based on secondary data routinely collected by public and publicly contracted health services within the SUS. The analysis focused on hospitalizations and deaths due to DM and hypertension from 2014 to 2024, using the state of Paraná as the unit of analysis. The study was reported in accordance with the Reporting of Studies Conducted Using Observational Routinely‐Collected Health Data (RECORD) guidelines (Benchimol et al. 2015).

### 2.2 Sample and Setting

Paraná is located in Southern Brazil and has an estimated population of 11.4 million inhabitants, a Human Development Index of 0.769, and 399 municipalities organized into 22 health regions (Instituto Brasileiro de Geografia e Estatística 2025). Primary health care serves as the main entry point to the health system and plays a central role in the prevention, follow‐up, and coordination of care for individuals with DM and hypertension (Soares et al. 2024).

### 2.3 Data Collection

Data were obtained from two national, publicly accessible health information systems. Hospitalization data were extracted from the Hospital Information System (SIH) of the SUS, which records inpatient admissions according to the International Classification of Diseases, 10th Revision (ICD‐10). Mortality data were retrieved from the Mortality Information System (SIM), which compiles death records classified by ICD‐10 codes. Data extraction was performed through the TabNet interface on November 27, 2025.

Hospitalizations were identified by place of residence, year of admission, and primary diagnosis, based on ICD‐10 morbidity groups. Hypertension was defined using codes I10–I15, including essential (primary) hypertension and other hypertensive diseases, while DM was defined using codes E10–E14. Mortality data were collected using standardized ICD‐10 grouping filters for DM and hypertensive diseases, as the considered system does not provide morbidity lists.

All datasets were exported in comma‐separated values format and consolidated for data cleaning and consistency checks. Records with missing or unknown information were excluded from inferential analyses. Annual hospitalization and mortality rates per 100,000 inhabitants were calculated using population estimates provided by the Brazilian Institute of Geography and Statistics, accessed through the Department of Informatics of the SUS.

### 2.4 Data Analysis

Analyses were; stratified by sex (male, female), age group (in years: 0–19, 20–39, 40–59, and ≥60), and for the total population (overall). Temporal trends were assessed using segmented log‐linear regression models with joinpoint analysis to estimate annual percent change (APC) and average annual percent change (AAPC), along with 95% confidence intervals. Positive values indicated increasing trends, negative values indicated decreasing trends, and values with confidence intervals including zero were classified as stable.

Annual rates were log‐transformed and modeled as dependent variables, with calendar year as the independent variable. Models were weighted by the standard errors of the rates and adjusted for first‐order autocorrelation when detected. All analyses were performed using the Joinpoint Regression Program, version 5.4.0.

For hypertension‐related hospitalizations among individuals aged 0–19 years, three calendar years showed zero admissions, which precluded trend estimation due to software constraints. For this age group, a three‐point moving average was applied to reduce random fluctuation and ensure statistical stability. Because this study used exclusively secondary, publicly available, anonymized data, with no individual identification or direct contact with human participants, ethical review was waived in accordance with Brazilian National Health Council Resolution No. 674/2022.

## 3 Results

Between 2014 and 2024, a total of 78,686 hospitalizations due to DM and 43,534 hospitalizations due to hypertension were recorded in Paraná. During the same period, 43,894 deaths due to DM and 39,758 deaths due to hypertension were registered. The average annual number of hospitalizations was 7,153 for DM and 3,957 for hypertension, while the average annual number of deaths was 3,991 for DM and 3,614 for hypertension. One death record due to DM with missing information on sex was excluded from the analysis.

### 3.1 Hospitalization and Mortality Rates for DM and Hypertension

Hospitalization rates for DM ranged between 75.0 and 55.0 per 100,000 inhabitants, with a slight decline after 2020. Mortality due to DM increased from approximately 30.0 per 100,000 inhabitants in 2014 to about 35.0 per 100,000 in 2024, indicating a modest overall increment. For hypertension, hospitalization rates varied between 40.0 and 30.0 per 100,000 inhabitants over the study period, showing reduction, whereas mortality rates reached almost 35.0 per 100,000 inhabitants (Figure 1).

**FIGURE 1:**
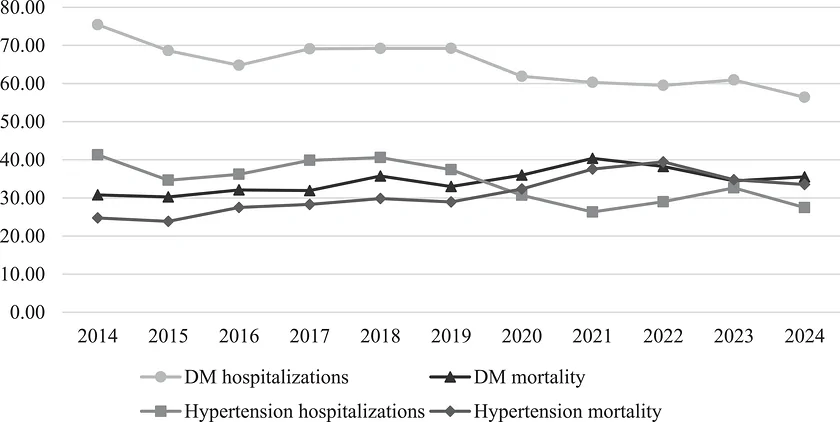
Time series of overall hospitalization and mortality rates due to diabetes mellitus and hypertension per 100,000 inhabitants, Paraná, Brazil, 2014–2024. DM, diabetes mellitus.

Hospitalization rates for DM declined among both male and female individuals, with a more pronounced reduction among female (from 82.17 to 53.65 per 100,000 inhabitants) and among individuals aged 60 years or older, whose rates decreased from 342.89 to 157.49 per 100,000 inhabitants. In contrast, mortality due to DM increased in both sexes, particularly among male (from 29.47 to 35.57 per 100,000 inhabitants). Across age groups, a proportional increase in mortality was observed among individuals aged 0–19 years, whereas a reduction was observed among those aged 60 years or older (Table 1).

**TABLE 1:** Hospitalization and mortality rates due to diabetes mellitus per 100,000 inhabitants, by sex and age group, Paraná, Brazil, 2014–2024.

Variable	2014	2015	2016	2017	2018	2019	2020	2021	2022	2023	2024
**Hospitalization**											
Sex											
Male	68.47	63.36	59.90	66.07	65.48	66.79	62.15	61.65	60.61	64.11	59.26
Female	82.17	73.70	69.52	72.05	72.80	71.56	61.65	59.03	58.48	57.94	53.65
Age group (years)											
0–19	15.77	17.64	17.87	18.02	19.03	20.68	20.29	21.32	23.47	26.41	25.04
20–39	21.56	20.80	18.77	22.64	26.75	27.33	25.48	26.25	27.79	31.32	31.59
40–59	92.71	81.05	74.15	78.52	73.28	73.25	66.03	61.77	59.17	56.34	56.12
≥60	342.89	297.16	275.59	281.55	271.81	259.05	217.91	204.89	191.47	188.38	157.49
**Mortality**											
Sex											
Male	29.47	29.34	30.57	31.69	34.66	32.22	35.17	38.85	37.32	33.78	35.57
Female	32.06	31.13	33.55	32.13	36.75	33.73	36.75	41.83	39.14	35.16	35.46
Age group (years)											
0–19	0.12	0.28	0.31	0.09	0.25	0.32	0.16	0.26	0.19	0.19	0.71
20–39	1.44	1.72	2.16	2.07	1.81	1.47	1.81	2.03	3.08	1.94	2.27
40–59	22.28	19.02	21.67	19.60	19.95	18.49	20.95	21.11	19.67	18.75	18.88
≥60	209.40	203.69	204.89	201.64	223.27	199.66	209.15	231.25	210.42	183.57	182.67

Hospitalizations due to hypertension decreased in both sexes, from 33.52 to 23.26 per 100,000 inhabitants among male and from 48.82 to 31.49 per 100,000 inhabitants among female. Declines were observed across all age groups, with the largest reductions among individuals aged 60 years or older (from 183.45 to 95.09 per 100,000 inhabitants). Conversely, mortality due to hypertension increased among both male and female individuals, reaching 33.37 and 33.69 per 100,000 inhabitants, respectively. Increases were also observed among individuals aged 20–39 years and 40–59 years (Table 2).

**TABLE 2:** Hospitalization and mortality rates due to hypertension per 100,000 inhabitants, by sex and age group, Paraná, Brazil, 2014–2024.

Variable	2014	2015	2016	2017	2018	2019	2020	2021	2022	2023	2024
**Hospitalization**											
Sex											
Male	33.52	28.98	30.28	31.89	32.32	29.79	25.07	21.25	25.76	28.02	23.26
Female	48.82	40.09	41.89	47.52	48.55	44.73	36.08	31.17	32.08	37.06	31.49
Age group (years)											
0–19	3.29	2.30	3.29	3.29	3.22	2.90	2.24	1.52	2.01	1.95	2.24
20–39	14.29	11.26	13.90	16.50	19.07	19.91	18.83	14.88	14.02	11.72	12.66
40–59	56.29	43.47	43.23	46.16	46.10	41.56	32.43	28.48	31.27	35.73	29.00
≥60	183.45	158.92	157.69	167.77	162.29	141.29	110.11	93.71	104.39	121.51	95.09
**Mortality**											
Sex											
Male	24.69	23.54	27.83	28.12	28.62	28.40	32.32	37.14	38.86	34.66	33.37
Female	24.79	24.16	27.15	28.45	31.02	29.48	32.35	37.96	40.03	34.86	33.69
Age group (years)											
0–19	0.03	0.00	0.06	0.06	0.03	0.03	0.00	0.03	0.10	0.06	0.00
20–39	0.79	0.79	0.84	1.06	0.86	0.97	1.09	1.14	1.26	1.10	1.02
40–59	13.50	11.65	13.75	14.07	12.21	12.89	15.12	16.04	16.95	16.24	14.04
≥60	178.90	169.59	188.38	187.27	197.02	182.71	196.26	223.54	226.89	191.41	181.84

### 3.2 Hospitalization and Mortality Trends for DM and Hypertension

Trend analyses indicated decreasing hospitalization rates for DM among female, adults aged 40–59 years, individuals aged 60 years or older, and in the overall population. In contrast, increasing trends were observed among individuals aged 0–19 years and 20–39 years. Mortality due to DM showed initial increasing trends for both sexes, for individuals aged 60 years or older and for the total population until 2021–2022, followed by periods of stability. Among individuals aged 0–19 years, an increasing trend was observed in the final period (2022–2024) (Table 3).

**TABLE 3:** Temporal trends in hospitalization and mortality rates due to diabetes mellitus per 100,000 inhabitants, by sex and age group, Paraná, Brazil, 2014–2024.

Variable	Period	APC (95% CI)	Trend	AAPC (95% CI)	Trend
**Hospitalization**					
Sex					
Male	2014–2024	−0.77 (−2.34; 0.85)	Stable	−0.77 (−2.34; 0.85)	Stable
Female	2014–2024	−3.76 (−5.14; −2.43)	Decreasing	−3.76 (−5.14; −2.43)	Decreasing
Age group (years)					
0–19	2014–2024	4.86 (3.94; 5.84)	Increasing	4.86 (3.94; 5.84)	Increasing
20–39	2014–2024	4.38 (1.76; 7.46)	Increasing	4.38 (1.76; 7.46)	Increasing
40–59	2014–2024	−4.71 (−5.90; −3.57)	Decreasing	−4.71 (−5.90; −3.57)	Decreasing
≥60	2014–2024	−6.64 (−8.38; −4.94)	Decreasing	−6.64 (−8.38; −4.94)	Decreasing
Overall	2014–2024	−2.33 (−3.73; −0.93)	Decreasing	−2.33 (−3.73; −0.93)	Decreasing
**Mortality**					
Sex					
Male	2014–2021	3.89 (2.97; 5.71)	Increasing	1.95 (1.09; 2.80)	Increasing
	2021–2024	−2.46 (−8.12; 0.58)	Stable		
Female	2014–2022	3.27 (2.28; 8.11)	Increasing	1.04 (‐0.28; 3.02)	Stable
	2022–2024	−7.41 (−14.38; 0.66)	Stable		
Age group (years)					
0–19	2014–2022	−1.96 (−40.29; 14.31)	Stable	9.21 (−1.06; 17.19)	Stable
	2022–2024	68.21 (6.61; 137.46)	Increasing		
20–39	2014–2024	3.83 (−0.11; 8.44)	Stable	3.83 (−0.11; 8.44)	Stable
40–59	2014–2024	−0.77 (−2.06; 0.52)	Stable	−0.77 (−2.06; 0.52)	Stable
≥60	2014–2021	1.22 (0.06; 3.92)	Increasing	−1.18 (−2.36; 0.01)	Stable
	2021–2024	−6.57 (−14.05; −2.61)	Decreasing		
Overall	2014–2021	3.81 (2.75; 6.36)	Increasing	1.67 (0.61; 2.77)	Increasing
	2021–2024	−3.17 (−9.90; 0.46)	Stable		

Abbreviations: AAPC, average annual percent change; APC, annual percent change; CI, confidence interval.

For hypertension, hospitalization rates showed decreasing trends among men, adults aged 0–19, 40–59 and individuals aged 60 years or older. Individuals aged 20–39 years exhibited an increasing trend until 2019, followed by a decreasing trend through 2024. Mortality due to hypertension showed increasing trends until 2022 among both sexes and among individuals aged 20–39 years and 40–59 years, with subsequent stabilization. Among individuals aged 60 years or older, a decreasing trend was observed in the final period (2022–2024) (Table 4). For overall population, an incresead AAPC was observed.

**TABLE 4:** Temporal trends in hospitalization and mortality rates due to hypertension per 100,000 inhabitants, by sex and age group, Paraná, Brazil, 2014–2024.

Variable	Period	APC (95% CI)	Trend	AAPC (95% CI)	Trend
**Hospitalization**					
Sex					
Male	2014–2024	−3.09 (−6.02; −0.17)	Decreasing	−3.09 (−6.02; −0.17)	Decreasing
Female	2014–2024	−3.95 (−8.83; 0.93)	Stable	−3.95 (−8.83; 0.93)	Stable
Age group (years)					
0–19	2014–2024	−5.20 (−10.80; −0.01)	Decreasing	−5.20 (−10.80; −0.01)	Decreasing
20–39	2014–2019	10.96 (6.37; 18.41)	Increasing	−0.60 (−2.78; 1.47)	Stable
	2019–2024	−10.97 (−16.83; −7.18)	Decreasing		
40–59	2014–2024	−5.72 (−9.66; −1.95)	Decreasing	−5.72 (−9.66; −1.95)	Decreasing
≥60	2014–2024	−6.12 (−9.80; −2.45)	Decreasing	−6.12 (−9.80; −2.45)	Decreasing
Overall	2014–2024	−3.60 (−8.07; 0.83)	Stable	−3.60 (−8.07; 0.83)	Stable
**Mortality**					
Sex					
Male	2014–2022	6.10 (4.36; 18.73)	Increasing	3.54 (1.65; 6.64)	Increasing
	2022–2024	−6.11 (−15.50; 3.69)	Stable		
Female	2014–2022	6.37 (5.36; 8.35)	Increasing	3.49 (2.43; 4.75)	Increasing
	2022–2024	−7.27 (−12.65; 0.00)	Stable		
Age group (years)					
0–19	2014–2024	5.17 (−3.06; 15.51)	Stable	5.17 (−3.06; 15.51)	Stable
20–39	2014–2022	5.82 (4.69; 8.44)	Increasing	2.84 (1.57; 4.47)	Increasing
	2022–2024	−8.29 (−14.80; 0.67)	Stable		
40–59	2014–2024	2.42 (0.64; 4.39)	Increasing	2.42 (0.64; 4.39)	Increasing
≥60	2014–2022	3.17 (2.11; 6.10)	Increasing	0.45 (−0.81; 2.05)	Stable
	2022–2024	−9.75 (−16.30; −1.84)	Decreasing		
Overall	2014–2022	6.24 (4.97; 11.54)	Increasing	3.52 (2.00; 5.67)	Increasing
	2022–2024	−6.68 (−14.46; 2.53)	Decreasing		

Abbreviations: AAPC, average annual percent change; APC, annual percent change; CI, confidence interval.

## 4 Discussion

The present study identified a sustained reduction in hospitalization rates due to DM and hypertension in Paraná between 2014 and 2024 across most population strata. Although with different magnitudes, this decline in hospitalizations is consistent with trends reported in other Brazilian states, such as Rio Grande do Sul (Stahnke et al. 2023) and Bahia (Silva et al. 2024), and has often been associated with the expansion and consolidation of primary health care. In contrast, mortality rates due to both conditions increased over much of the study period, particularly among younger and middle‐aged adults.

Time‐trend analyses reinforced this apparent paradox. Hospitalizations due to DM showed decreasing trends in the overall population, especially among individuals aged 60 years or older, while mortality exhibited initial increasing trends that stabilized only in more recent years. A similar pattern was observed for hypertension, with declining hospitalization rates accompanied by increasing mortality until 2022. This dissociation suggests that reduced hospital admissions do not necessarily reflect a lower disease burden and may instead indicate delayed access to care or changes in hospitalization thresholds, leading to more severe outcomes outside the hospital setting.

The concurrent reduction in hospitalizations and increase in mortality may reflect a shift toward compression of morbidity, whereby individuals experience fewer hospital admissions but with greater severity and lethality (Stahnke et al. 2023; Silva et al. 2024). Additionally, the COVID‐19 pandemic likely acted as a disruptive factor in these time series, affecting health care utilization, delaying diagnosis and follow‐up of chronic conditions, and producing cohort effects in the post‐pandemic period. These disruptions may have disproportionately affected adults of working age, contributing to the increases in mortality among younger populations (Du et al. 2024; Törő et al. 2025).

Another plausible explanation relates to persistent gaps in early detection and longitudinal management of DM and hypertension. Underdiagnosis and inadequate follow‐up can allow these conditions to progress silently, increasing the risk of acute complications and premature death. Despite the central role of primary health care in chronic disease management, barriers such as limited access, fragmented care pathways, and socioeconomic inequalities may undermine effective control, particularly among younger adults who traditionally have lower engagement with selfcare and health services (Macedo et al. 2025).

For example, in Brazil, a population‐based cross‐sectional study using data from the National Health Survey, with a representative sample of urban and rural areas and including 10,460 adolescents and young adults, reported high prevalences of behaviors such as smoking (8.9%), monthly alcohol consumption (28.7%), and binge drinking episodes (18.5%). These behaviors reinforce early exposure to cardiovascular and metabolic risks, signaling a future scenario of increasing burden of DM and hypertension (Pedroso et al. 2023).

From a public health perspective, the findings underscore the importance of interpreting declining hospitalization rates with caution. While reductions in potentially avoidable hospitalizations are often viewed as indicators of improved primary care performance, they should be examined alongside mortality trends and other outcome measures. The observed divergence between hospitalizations and mortality highlights the need for integrated monitoring strategies that capture both service utilization and population health outcomes.

This study has limitations inherent to the use of secondary data, including potential misclassification of diagnoses and reliance on routinely collected information. Nevertheless, the use of standardized national databases and robust time‐series methods strengthens the validity of the findings. By examining long‐term trends in both hospitalization and mortality, this study contributes to a more comprehensive understanding of the evolving burden of DM and hypertension and highlights critical challenges for chronic disease management within health systems.

### 4.1 Implications for Public Health Nursing Practice

The divergent trends observed between declining hospitalizations and increasing mortality from DM and hypertension highlight the strategic role of public health nurses in strengthening active surveillance within primary health care. Nurses are uniquely positioned to identify individuals at risk through routine contacts, community outreach, and population‐based risk stratification, particularly among younger and middle‐aged adults who often have lower engagement with health services. Active surveillance led by nurses—integrating screening, early detection, and follow‐up—can mitigate delays in diagnosis and interrupt trajectories that culminate in preventable mortality, even in contexts where hospital admissions appear to be declining.

Also, the integrated use of hospitalization and mortality data offers important opportunities for strengthening nursing practice in public health surveillance and health equity. Routine analysis of data from systems can inform nurse‐led planning, identify vulnerable subpopulations, and guide targeted interventions at the community level. By bridging epidemiologic surveillance with frontline care, public health nurses serve as a critical link between prevention, continuous care, and equity‐oriented strategies, ensuring that improvements in service utilization translate into meaningful gains in population health outcomes.

## 5 Conclusions

Overall, the findings demonstrate that declining hospitalization rates, when considered in isolation, do not adequately reflect improvements in population‐level outcomes related to DM and hypertension. The coexistence of reduced hospitalizations and increased mortality highlights the limitations of using health service utilization as a proxy for disease control and suggests that changes in disease severity, timely diagnosis, and prevention of fatal complications may not be fully captured. These results emphasize the need for integrated interpretation of hospitalization and mortality trends in population‐based epidemiologic studies of chronic diseases.

## Declaration of Generative AI and AI‐Assisted Technologies in the Manuscript Preparation Process

During the preparation of this work, the authors used ChatGPT (OpenAI) to support language editing and text organization. After using this tool, the authors reviewed and edited the content as needed and take full responsibility for the content of the published article.

## Funding

This research did not receive any specific grant from funding agencies in the public, commercial, or not‐for‐profit sectors.

## Conflicts of Interest

The authors declare that they have no competing interests.

## Data Availability

The data that support the findings of this study are available from the corresponding author upon reasonable request.
